# Effect of dabrafenib on melanoma cell lines harbouring the BRAF^*V600D/R*^ mutations

**DOI:** 10.1186/1471-2407-13-17

**Published:** 2013-01-14

**Authors:** Giusy Gentilcore, Gabriele Madonna, Nicola Mozzillo, Antoni Ribas, Antonio Cossu, Giuseppe Palmieri, Paolo A Ascierto

**Affiliations:** 1 Department Melanoma, Istituto Nazionale Tumori Fondazione Pascale, Naples, Italy; 2 Department of Medicine, Jonsson Comprehensive Cancer Center, UCLA, Los Angeles, CA, USA; 3 Department of Pathology, Hospital-University Health Unit (AOU), Sassari, Italy; 4 Institute of Biomolecular Chemistry, National Research Council (ICB-CNR), Sassari, Italy; 5 Unit of Medical Oncology and Innovative Therapy, Istituto Nazionale per lo Studio e la Cura dei Tumori “Fondazione G. Pascale”, Via Mariano Semmola, 80131, Naples, Italy

**Keywords:** BRAF inhibitor, Dabrafenib, Growth inhibition, Melanoma therapy

## Abstract

**Background:**

Conventional therapeutic agents are largely unsatisfactory into the treatment of malignant melanoma. Recently, an innovative approach based on inhibitors of the mutated BRAF gene (which represents the most prevalent alteration in melanoma patients) appears very promising from the clinical point of view. On this regard, a new compound, dabrafenib (GSK2118436), has been demonstrated to be effective in patients carrying the BRAFV600E/K mutations. We here tested dabrafenib for its capability to inhibit cell growth on primary melanoma cell lines, established from patients' tumour tissues and carrying the BRAFV600D/R mutations.

**Methods:**

Three melanoma cell lines were tested: M257 wild-type BRAF, LCP BRAFV600R and WM266 BRAFV600D. The MTT assays were performed using standardized approaches. To evaluate the inhibition of MAPK pathway and the consequent inhibition of cellular proliferation, the phosphorylation of ERK was examined by Western Blot analysis performed on total protein extracts from cell lines after treatment with dabrafenib.

**Results:**

Our experiments demonstrated an effective action of Dabrafenib (GSK2118436) and the inhibition of MAPK pathway in melanoma cell lines carrying BRAFV600D/R mutations.

**Conclusion:**

These results could be helpful to enlarge the number of melanoma patients who may benefit of a more effective targeted treatment.

## Background

The mitogen-activated protein kinase (MAPK) pathway is a key regulator of cell cycle progression, commonly activated in human tumours through somatic oncogenic mutations in *RAS*, *RAF*, and *MEK* genes [1].

In melanoma, the most commonly mutated component of the MAPK pathway is the *BRAF* gene; among others, the most prevalent *BRAF* mutation (nearly, 90% of cases) is represented by a substitution of valine with glutamic acid at position 600 (V600E) [2]. This amino acid change leads to oncogenic activation of BRAF, with an increase of its kinase activity, and subsequent induction of phosphorylation of the downstream ERK protein. Constitutively activated ERK then stimulates cell proliferation and survival, sustaining tumour maintenance and growth [2]. The remaining *BRAF* mutations are mostly represented by other V600 subtypes (V600K, V600D, and V600R), which account for about 8% of the pathogenetic gene sequence variants [3]. In our experience, mutations in *BRAF* gene occur in 43% of primary melanomas and 48% of metastatic melanomas [4].

A significant benefit in melanoma treatment has been recently achieved with two selective inhibitors: vemurafenib (PLX4032), which seems to particularly act on BRAF^V600E^ mutants (although it has been demonstrated to also inhibit proliferation of melanoma cell lines expressing other codon 600 BRAF mutations: V600D, V600K, and V600R) [5-7], and dabrafenib (GSK2118436), which has been demonstrated to mostly inhibit the kinase activity in BRAF^V600E/K^ mutants [8,9].

To investigate whether this latter compound may exert inhibiting effects on a wider range of BRAF mutants (similarly to those previously reported with vemurafenib/PLX4720 [5,7]), melanoma cell lines carrying the two remaining most prevalent *BRAF* mutations (V600D and V600R) were here treated with dabrafenib and cellular proliferation was then assessed.

## Methods

To determine effects on proliferation, melanoma cell lines were treated in triplicate with increasing concentrations (3 to 100 nM) of dabrafenib for 72 hrs. To evaluate the occurrence of inhibitory effects on the ERK activation, a Western Blot analysis was performed on total proteins extracted from cell lines after the 72-hrs treatment with the BRAF inhibitor. In particular, the rate of phosphorylated ERK was estimated on equal amounts of total protein for cell lysates; GAPDH was used as an internal control for total protein expression levels. Cell doubling time was determined from cell numbers measured in duplicates every 24 hours for a period of 7 days, using Burker cell counts.

## Results and discussion

**Figure 1:**
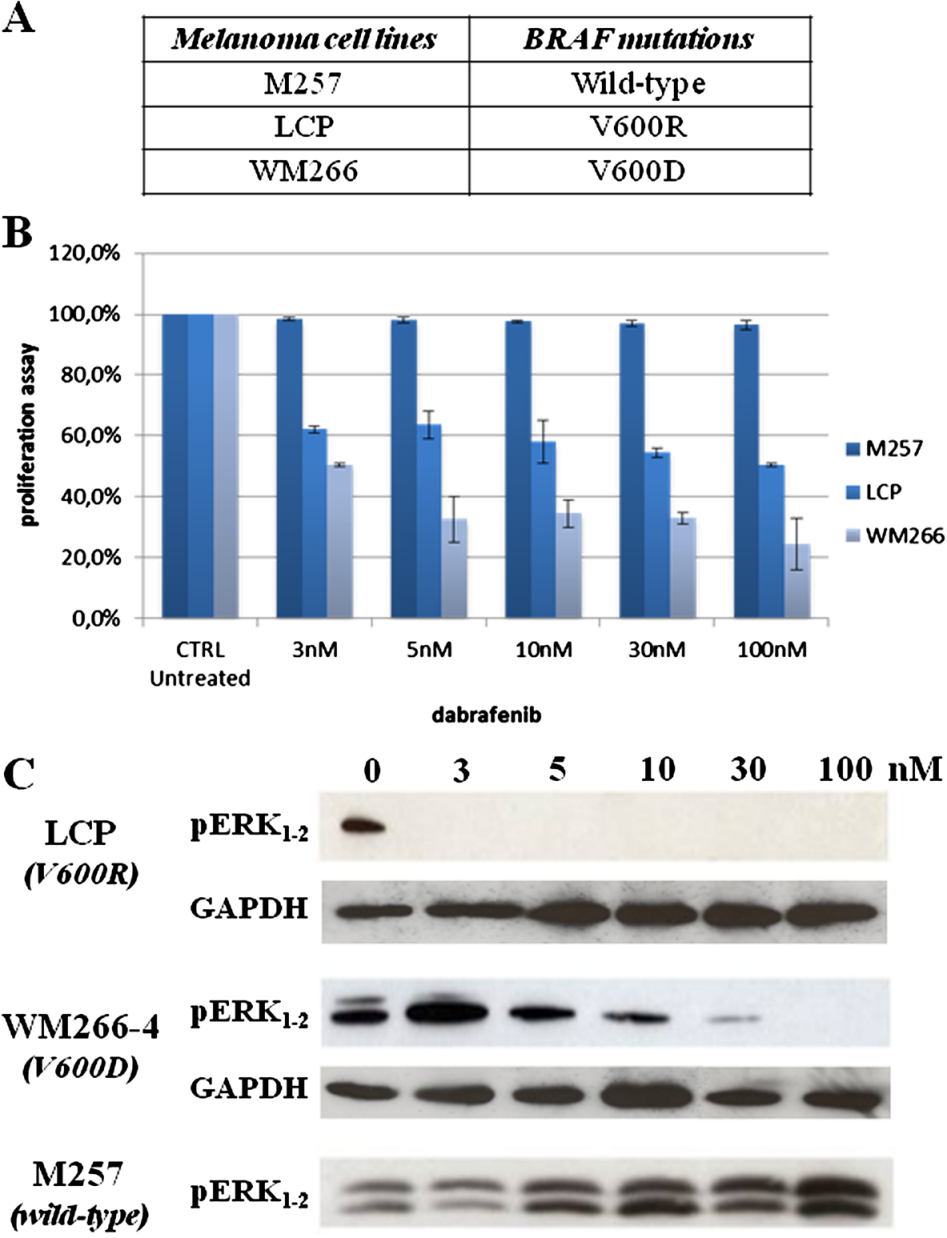
**Effects of dabrafenib in melanoma cell lines.A***. BRAF* mutational status in our series of melanoma cell lines. **B***.* Antiproliferative activity of increasing concentrations of dabrafenib in each cell line, as compared with the untreated (0) control. **C***.* Inhibitory effects of dabrafenib on ERK_1-2_ phosphorylation, under the same experimental conditions (drug concentrations and time of treatment) as above.

Using a panel of melanoma cell lines derived from the establishment of excised primary and metastatic tumours, we have investigated the ability of dabrafenib to both exert an antiproliferative activity on cultured melanoma cells and block the ERK signalling induced by the mutated BRAF. In our assay, the LCP melanoma cell line carried the BRAF^V600R^ mutation, whereas the WM266 melanoma cell line presented the BRAF^V600D^ variant (Figure 1A); the M257 melanoma cell line, with a wild-type *BRAF*, was used as control*.*

In our series, dabrafenib showed a remarkable inhibition of cell proliferation in both melanoma cell lines carrying a mutated *BRAF* (regardless of the type of mutation, BRAF^V600D^ or BRAF^V600R^), with lack of significant antiproliferative effects in control cells presenting a wild-type *BRAF* (Figure 1B). Regarding the interference with the ERK signaling, cell lines with BRAF^V600D/R^ mutations presented a faster and stronger inhibition of phosphorylated ERK as compared to control cells with a wild-type *BRAF* (Figure 1C).

Dabrafenib has been reported to be specifically active on BRAF^V600E/K^ mutants; data here presented strongly suggest that BRAF^V600D/R^ mutations might be also included as an effective target of the drug. Nevertheless, although the BRAF^V600D/R^ mutations constitute a small fraction of the entire set of oncogenic variants in *BRAF* gene, the availability of a drug showing effectiveness on a more inclusive variety of BRAF-mutated targets could be helpful from the practical point of view, allowing clinicians to enlarge the subset of melanoma patients who may benefit of a more effective targeted treatment.

## Conclusions

Although carried out on a limited series of melanoma cell lines, our study provided evidence that dabrafenib may exert a wider inhibitory activity on oncogenic variants of *BRAF*.

## Abbreviation

MAPK: Mitogen-activated protein kinase.

## Competing interests

Dr Paolo A. Ascierto participated to Advisory Board from Bristol Myers Squibb, MSD, Roche Genentech, GSK, and received honoraria from Brystol Myers Squibb, MSD and Roche-Genentech. All remaining authors declare the absence of any Competing Interest.

## Authors’ contributions

GG performed data acquisition, data analysis, preparation of the illustration, and drafted the manuscript; GM contributed in data analysis and draft of the manuscript; NM contributed in interpretation of the data; AR critically revised the manuscript; AC contributed in data analysis; GP collaborated in the draft of the manuscript; PAA conceived and supervised the study, and revised the manuscript. All authors read and approved the final version of the manuscript.

## Pre-publication history

The pre-publication history for this paper can be accessed here:

http://www.biomedcentral.com/1471-2407/13/17/prepub
